# Molecule Clustering Dynamics in the Molecular Doping Process of Si(111) with Diethyl-propyl-phosphonate

**DOI:** 10.3390/ijms24086877

**Published:** 2023-04-07

**Authors:** Mattia Pizzone, Maria Grazia Grimaldi, Antonino La Magna, Silvia Scalese, Jost Adam, Rosaria A. Puglisi

**Affiliations:** 1 Consiglio Nazionale delle Ricerche (CNR) Istituto per la Microelettronica e Microsistemi (IMM), Strada VIII n.5, Zona Industriale, 95121 Catania, Italy; 2 Dipartimento di Fisica e Astronomia “Ettore Majorana”, Università degli Studi di Catania, Via S. Sofia, 64, 95123 Catania, Italy; 3 Computational Materials Group, Centre for Photonics Engineering, Mads Clausen Institute, University of Southern Denmark (SDU), 5230 Odense, Denmark

**Keywords:** self-assembly, molecular doping, liquid phase deposition, nucleation, surface treatments, sheet resistance, nucleus capture zone

## Abstract

The molecular doping (MD) process is based on the deposition of dopant-containing molecules over the surface of a semiconductor substrate, followed by the thermal diffusion step. Previous studies suggest that, during the deposition, the molecules nucleate clusters, and at prolonged deposition times, they grow into self-assembled layers on the sample to be doped. Little is known about the influence of nucleation kinetics on the final properties of these layers and how they change when we modify the solution properties. In this work, we examine the nucleation rate and the molecular surface coverage kinetics of diethyl-propyl phosphonate on silicon at different solution concentrations and how these conditions influence the final electrical properties of the doped samples. We present a high-resolution morphological characterization of the as-deposited molecules together with the electrical results of the final doped samples. The experimental results show a non-obvious behavior, explained through understanding of the competition between the molecules’ physisorption and chemisorption mechanisms. As a consequence, due to the deeper knowledge of the deposition phase, a finer tuning of the conductive properties of MD-doped samples is achieved.

## 1. Introduction

Most semiconductor devices are fabricated by the introduction of dopant impurities inside the material that are used to modify its electrical properties. The act of introducing those impurities is called doping. Common doping methods (ion implantation and diffusion-based techniques) can cause a plethora of issues related to safety, crystal lattice damage, and cost, especially for the fabrication of nanoscale devices with non-planar architectures. Molecular doping (MD) has been introduced as a low-cost alternative to conventional doping methods [1,2,3,4,5,6]. The technique consists of the deposition of dopant-containing precursor molecules from the liquid phase on the samples to be doped. The peculiarity of MD relies on the fact that the molecules containing the dopant atoms self-assemble on the substrate surface [1]. The literature on self-assembled monolayers (SAM) is vast, and the applications, at the laboratory level, have been demonstrated in many cases, such as in Li-batteries [7], transparent conductive oxides [8], nonlinear optics [9], and more extensively in biofunctionalized systems [10]. SAMs have also been applied to modify the electronic properties of the surface, where the SAM element represents the active dopant component [11,12,13]. In contrast to the last case, the molecules represent the carrier for the dopant atom, not the active dopant element itself in the MD process. For this reason, many of the technical requirements that give MD industrial potentiality are relaxed with respect to the previously cited approach. Since the deposition step is made in liquid solutions, the resulting doping profile is conformal, i.e., it follows the shape of the sample surfaces from micro-sized systems [14] to nano-sized ones [15,16], with respective curvature radii of roughly 1.75 μm, 50 nm, and 5 nm. Moreover, the possibility to produce highly doped ($10^{20}$ ${\mathrm{cm}}^{-3}$) metallurgical junctions with a thickness of 5–10 nm has been demonstrated, which makes the technique versatile and applicable to many geometries [2,17,18,19]. Depending on the application, the desired doping dose in the Si industry spans within the wide range of 10${}^{12}$–10${}^{18}$ ${\mathrm{cm}}^{-2}$. For this reason, control of MD efficiency has been explored thoroughly in the literature. Several studies have been conducted on dopant dose control through the coating time [1,4], the molecular footprint [1,5,20], the post-deposition surface cleaning process [21] or through diluting the deposition solution with a neutral molecule [22], i.e., not participating in the doping process. Studies of self-assembled molecular layer nucleation have observed how molecules can diffuse on the surface and how they tend to form ordered structures [23,24,25]. This is further supported by a study on how the molecule sticks to the substrate, through which bonds and orientation [16,21].

Although the kinetics of molecular deposition and its relation to the deposition solution parameters have been studied in the past [4,5,26,27,28,29,30,31], an in-depth study of the effects of solution concentration on the final electrical properties of samples has not yet been conducted. The present work shows the morphological evolution of the diethyl-propyl-phosphonate (DPP) deposition on Si over time at several solution concentrations, the monolayer formation, and their comparison with the electrical performance of doped samples. We present these data together with a study of monolayer formation evolution by analyzing surface coverage data with the Avrami function, which describes the temporal behavior of phase changes, and we provide curve fitting on the collected data, which can give insight into the behavior of the DPP-Si system at a molecular level [32,33,34].

## 2. Results and Discussion

**Figure 1:**
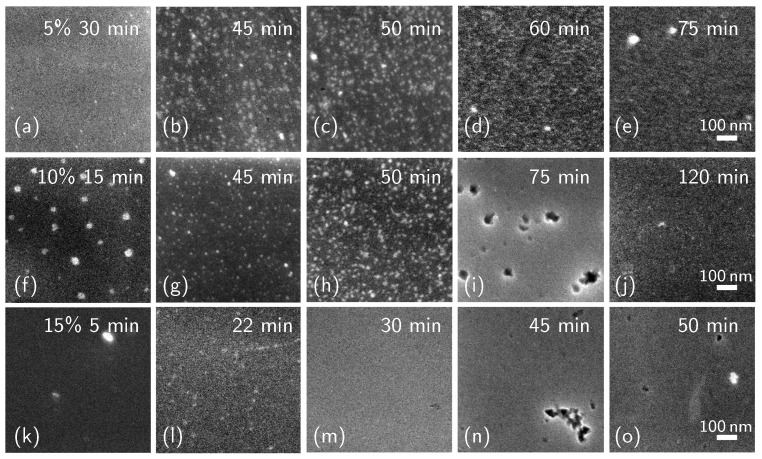
SEM images of the samples at different deposition times. Each row of images refers to samples dip-coated in solutions of different concentrations: (**a**–**e**) 5%; (**f**–**j**) 10%; (**k**–**o**) 15%. The scale bars refer to all the figures.

We carried out the deposition step in a solution of DPP and mesitylene at different concentrations: 5%, 10%, and 15% (*v*/*v*). We kept the samples inside the solution at a constant temperature between 7 and 120 min. The samples were then divided into two groups: one group was subjected to a surface cleaning process consisting of a rinse in acetone, alcohol, and water, and 15 min of stirring in acetone (the “cleaned” samples), while the other group was not (the “as-deposited” samples). We analyzed the last samples with scanning electron microscope (SEM) imaging. We selected SEM microscopy, which in our case, exploited a field emission gun-based instrument providing high-resolution images, because other techniques were not able to provide high-resolution morphological information on how the molecules are arranged and clustered. Figure 1 shows several SEM micrographs of the as-deposited samples for each series at different deposition times: Figure 1a–e refer to samples produced in a 5% concentrated solution (5% samples), Figure 1f–j are samples obtained with a 10% concentrated solution (10% samples), Figure 1k–o show samples dip-coated in a 15% concentrated solution (15% samples).

As can be seen from the images in Figure 1, the molecular surface coverage of the three series of samples increases over time for all three solution concentrations explored. A nucleation phenomenon can be observed in Figure 1a–d, f–h, k and l, before the impingement between the clusters and full coverage occurs. From a preliminary observation of the images, it is clear that the nucleation and impingement appear to be heavily influenced by the concentration of the solution in which the deposition happens.

**Figure 2:**
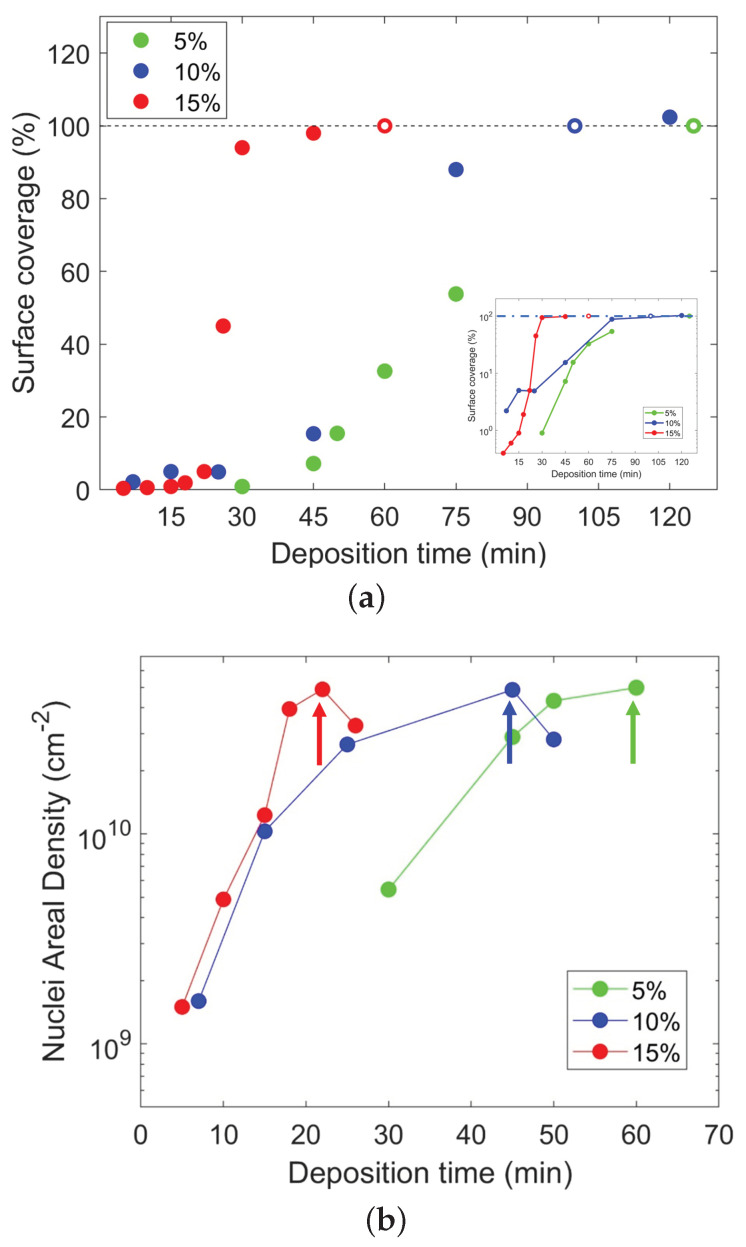
(**a**) Surface coverage as a function of deposition time. The dashed line indicates 100% coverage. Coverage higher than 100% refers to full monolayer coverage plus additional coverage deriving from forming a second layer on top of the first. (**b**) Nuclei areal density of the samples vs. deposition time. The arrows indicate the nuclei density maximum value, after which there is a decrease due to the coalescence effect.

To be quantitative, we measured the surface coverage using the image analysis method described in the Materials and Methods section. We report the results in Figure 2a as a function of the deposition time. For the 5% samples (green symbols), the surface coverage increases from 0.9% after a 30 min deposition to 63.8% after a deposition of 75 min; for the 10% samples (blue symbols), the measured surface coverage is between 2.2% at 7 min and a complete monolayer for deposition of 120 min; while for the 15% samples (red data), the surface coverage is 0.4% at 5 min and rises to an almost complete layer at 50 min. The dashed line indicates 100% coverage. Coverage higher than 100% refers to full monolayer coverage plus additional coverage deriving from forming a second layer on top of the first.

From the same image analysis, we also measured the nuclei surface density. We collected the results shown in the graph of Figure 2b, where the molecular nuclei areal density vs. deposition time is represented for the 5% (green), 10% (blue), and 15% (red data) concentrations. The arrows indicate the apparent decrease in nuclei density due to the impingement effect.

For the successive part of the discussion, it is essential to note that: (i) the surface coverage increases more rapidly when the concentration increases; (ii) for the high concentrations, the rate decreases during a long deposition time, i.e., when the surface coverage reaches values larger than 80%; (iii) the nucleation density and rate strongly depend on the solution concentration; and (iv) they increase with this parameter.

Once the nuclei density behavior for several solution conditions was investigated, we intended to study the electrical properties of the final doped samples. For a deep comparison between the morphological characteristics and the sheet resistance results, however, it is essential to recall previous knowledge on the interface between the molecules and the substrate. In a previous work, we demonstrated how the molecular layers are constituted by molecules chemically and physically bonded to the substrate through, respectively, covalent and Van der Waals bonds [21].

In particular, at the end of the process, molecules of the first assembled layer are mostly chemically bonded to the Si surface. In contrast, molecules of the second layer are physically bonded to the first. Both molecules’ layers contribute to the samples’ electrical properties, but the last ones are easily removed from the surface after chemical treatment. It is then essential to evaluate how the changes in the chemically and physically bonded molecule populations reflect the electrical properties of the samples. Consequently, it is necessary to study the samples’ properties by removing the physisorbed molecules and comparing the results with the physi- and chemisorbed cases. For this reason, we investigated the samples after chemical treatment to remove the physisorbed component.

**Figure 3:**
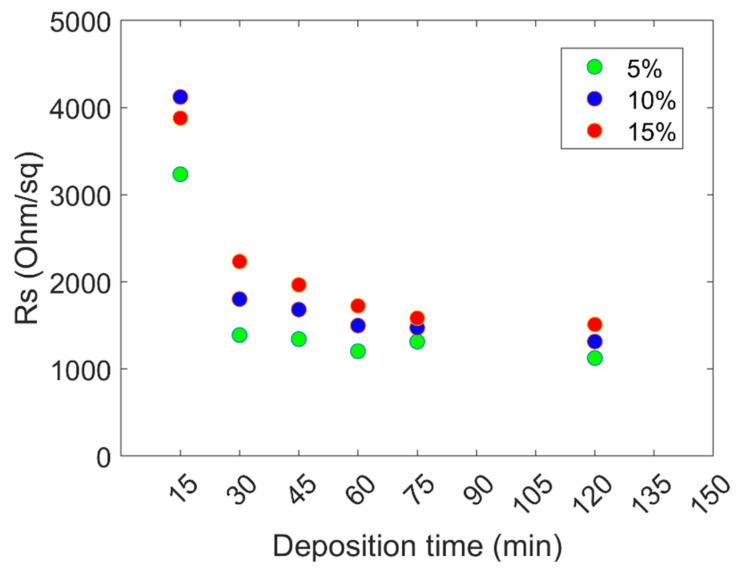
Sheet resistance data of the the 15% (red), 10% (blue) and 5% (green) sample series.

The data on the treated samples are reported in Figure 3, showing sheet resistance for the 15% (red), 10% (blue), and 5% (green) cases. The data evidence that the sheet resistance (Rs) systematically decreases with deposition time, as expected, but also when the concentration decreases, i.e., the samples grown with diluted solutions exhibit better electrical conductivity. This contrasts the morphological data, showing faster molecule coverage growth for the high solution concentrations. Always in apparent contrast with the morphological data is the fact that at deposition times corresponding to full or almost full coverage of the surface with the molecules (120 min for the 5%, 80 min for the 10%, and 30 min for the 15%), the Rs values are still in a decreasing trend. Another aspect is that the sheet resistance difference among the three curves decreases as the deposition time increases, trending roughly to a common saturation value for the three solutions. Nevertheless, at prolonged deposition times, there are still measurable differences between R values, with the diluted solution still showing the lowest resistance among the three. These results show that the solution concentration affects the electrical properties counterintuitively, and the way the molecules interact with the sample surface must be deeply understood.

To shed further light on the deposition process in the three cases, we use the Avrami function because it provides information on the dimensionality of the nucleation process of the molecule clusters.

**Figure 4:**
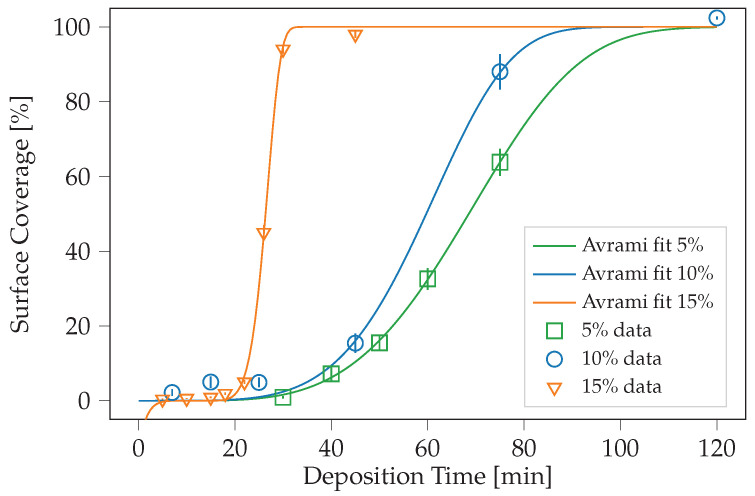
Graph of the Avrami fits for the three sets of data: 5% in green, 10% in blue, and 15% in orange.

We fit the surface coverage data to the Avrami function [32,33,34], defined by

(1) $$\begin{array}{c} F\left(t;t_{0},k,n\right)=1-\exp\left(-{\left(\frac{t-t_{0}}{k}\right)}^{n}\right), \end{array}$$

where t>0 is the incubation time, k is related to the activation energy, and *n* is the Avrami exponent, associated with the dimensionality of the nucleation process [32,33,34]. The *n* exponent in the classical Avrami theory is an important parameter and represents the reason why we chose to use this theory to fit our data: *n* is equal to d + 1, where d is the dimensionality of the phenomenon; thus, the 2D phase changes have an exponent of 3, and the 3D ones have *n* = 4 [35]. When *n* is larger than 4, the literature refers to transient and nonlinear behaviors [36,37,38,39], which represent the key elements to understanding the data that will be discussed below. We used the weighted least squares fitting for this task. We summarize the resulting parameters in Table 1 and showcase the functional fits in Figure 4. The very low value of $t_{0}$ in the 10% case could result from the process fluctuations at the early deposition stages. Moreover, despite the use of a high-resolution field emission gun-SEM microscope to acquire the images, the data collected through microscopy could not be accurate at the initial deposition stages for the following reasons: (i) image resolution; (ii) inhomogeneity of the sample surface; (iii) noise in the imaging process.

The *n* trend for the three series suggests a modification in the MD deposition nature. As said, the *n* exponent predicts the phenomenon dimensionality. For *n* larger than 4, we know that it is the case of transient nonlinear processes. In our case, *n* ranges between 3.86 and 6.66, increasing with concentration, suggesting that these transient behaviors are more prevalent at high solution concentrations. In the case of nonlinear behaviors, two main types of deposition mechanisms have been evidenced in the literature [40,41]: growth by direct adsorption, in which the deposited species directly develop a strong chemical bond with the deposited surface upon impinging on it, and growth via an adsorbed layer, in which an intermediate state of physisorption is involved between the desorbed and chemisorbed states. Our previous study shows that both sticking mechanisms are possible at the surface, but this study was experimentally performed at a specific solution concentration, and the simulations were performed on a small number of molecules [16,21]. Nothing is known thus far when the concentration varies and how large numbers of molecules aggregate. By considering these possible deposition and bonding mechanisms, an interpretation of the morphological data of Figure 2 and their relation to the sheet resistance of Figure 3 can be given. With a 5% solution concentration, the resulting Avrami exponent roughly matches the classical prediction of *n* = 3 for two-dimensional growth. Even though the surface coverage is relatively low compared to the other cases at the same deposition time, the molecules develop chemical bonds upon impingement. They are not rinsed away by the surface treatment. In the 10 and 15% cases, the surface coverage follows a much steeper curve, with *n* = 4.94 and 6.66, respectively, indicating a change in how the molecules are deposited. The sheet resistance data for the 15% cleaned sample series, higher than the other cases, suggest that physisorption is the bonding mechanism prevailing in this case. This is attributed to the high nucleation rate, which allows the physisorbed molecules to cover the surface with faster dynamics compared to those needed for the chemisorption. Thus, at high concentrations, physisorption is the dominant mechanism in physi-/chemisorption competition.

To understand why the sheet resistance at the corresponding deposition times is still decreasing, with high molecular coverage for the 80% and above cases in Figure 1 and Figure 2a, we have to take into account the spatial distribution of the molecule clusters and how the new molecules adhere to the substrate in the vicinity of a grown cluster. At high deposition times, when the coverage is large, another mechanism takes place, which can be explained by introducing the concept of the nucleus capture zone [42,43,44,45,46,47,48,49]: molecules impinging on the surface around one nucleus are most likely to be captured by that nucleus and, thus, contribute to its growth. Each capture zone is approximated by the surface points closer to the nucleus’ center of mass than to other nuclei. Each nucleus’ capture zone depends, as a consequence, on the nuclei population around it [50]. This means that the faster the nucleation rate (high concentrations), the larger the total perimeter of the clusters formed on the substrate.

**Figure 5:**
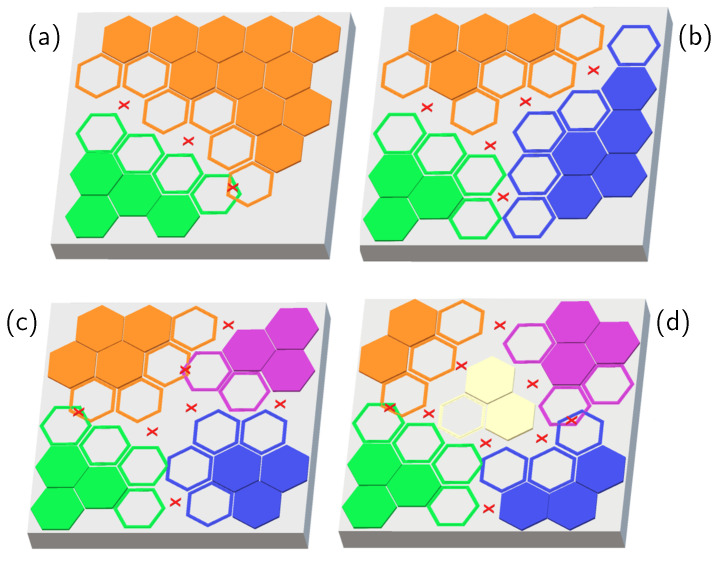
A schematic of the relationship between nuclei surface density and the surface zones available for chemisorption. In the picture, a chemisorbed molecule is represented by a fully colored shape. In contrast, the empty structure represent the surface spots where the depositing molecules could chemisorb when captured by a nucleus, identified by its color. Marked areas are either overlaps between incompatible chemisorption sites or are unavailable for developing a chemical bond with a depositing molecule. The schematic shows that the available surface diminishes with more nuclei occupying the same surface portion. Examples with (**a**) two, (**b**) three, (**c**) four, and (**d**) five nuclei.

By nucleation and successive growth, molecular clusters keep forming on the sample surface. During this growth, the areas enclosed between the edges of these molecular clusters can present bonding sites in which the following deposited DPP molecule will not be chemisorbed but will be physisorbed. Figure 5 shows a process schematic. In the figure, a chemisorbed molecule is represented by a full-colored polygon. In contrast, the empty structures represent the surface areas where the depositing molecules can potentially be captured by a nucleus, identified by the same color, through chemisorption. The areas marked with crosses are either overlaps between incompatible chemisorption sites or are unavailable for developing a chemical bond with a depositing molecule. The schematic shows that the available surface decreases with the nuclei density increment—and then with a larger perimeter—occupying the same surface portion, i.e., for high nuclei density. Examples with (a) two, (b) three, (c) four, and (d) five nuclei are shown in the figure. The available space for chemisorption of the DPP molecules on the Si surface then depends on the nuclei surface density. The transient molecules’ physisorbed state during deposition from the concentrated solutions then has an impact on sheet resistance through the capture zone role: at high solution concentrations, and thus, high nucleation rates, the surface areas available for chemisorption are reduced, and as a consequence, the sheet resistance of the cleaned samples increases. The density of the molecules in the solution and arriving on the Si surface has an impact on the bonding mechanisms, changing the local minimum energy configuration, as predicted in our previous work: the most stable energy configuration has a minimum for the covalent bond, but an unstable Van der Walls bond can be formed as well [21,28]. In this frame, the apparent discrepancy between the morphological and electrical data is explained: when the nucleation rate is high, i.e., for high concentrations, physisorption is more favored than chemisorption, and the molecules are rinsed away through the chemical treatment. This happens consistently for all deposition times, although a complete monolayer is expected to be reached at different times for the different dilutions. When the coverage reaches a level where the spatial distribution plays a role (around 80% and above), the perimeter of the formed nuclei is high, and the capture zone plays a role in impeding/retarding the covalent bonding mechanism. This leads to a decrease in the chemisorption nucleation rate at high concentrations and a corresponding reduction in electrical efficiency. Together, with a scientific understanding, these results allow for better tuning of the doping process and surface functionalization controlled at nm sizes, which is vital for certain applications, specifically for device scaling.

## 3. Materials and Methods

The employed substrates were 1 × 1 ${\mathrm{cm}}^{2}$ 1–10 ohm/sq p-type <111> Si samples. The molecular deposition was performed right after the substrates were sonicated in acetone, alcohol, and milli-Q water for 5 min each and then cleaned in HF for 1 min. The deposition step was carried out in 5%, 10%, and 15% concentrated (*v/v*) solutions of DPP (Abcr, 95%) and mesitylene (Abcr, 98%+). Samples and solution were kept at the solution’s boiling point temperature for the desired time. Samples were divided into two groups: one was treated with a surface cleaning process consisting in rinsing in acetone, ethanol, and water and stirring in acetone with a magnetic stirrer for 15 min (“cleaned” samples), while the other group (“as-deposited” samples) was analyzed right after deposition.

SEM images were taken with a Zeiss Supra 35 FE-SEM (Oberkochen, Germany) with a primary energy beam of 3 keV. The surface coverage was extracted from these images using a noise reduction strategy based on fast Fourier transform (FFT) filtering through an applied bandpass mask [49]. The inverses of the filtered FFTs were then transformed back by an inverse FFT. The application of brightness threshold resulted in identification of the areas of interest and measurements of the surface coverage. Measurements and calculations were performed with the Digital Micrograph 3 software. The contrast was optimized to increase the signal-to-noise ratio. The image resolution allowed for the evaluation of objects down to 32 ${\mathrm{nm}}^{2}$ (or 6.4 nm in diameter if we assume circular cluster images). The developed image recognition process described above allowed for noise identification and treatment, proper choice of the object-identification threshold, and image transformation from grayscale to black and white.

Thermal diffusion processes were performed in $N_{2}$ environment brought to 1050 °C for 500 s, with an initial ramp of 10 °C per minute, starting from 600 °C. No oxide layer was deposited on the sample surfaces before the annealing process.

Four-point probe sheet resistance measurements were performed using a Keithley 237 source-measure unit. Measurements were performed in a dark environment to eliminate light-induced conductivity modifications on the samples. No metallic contacts were deposited on the specimens to improve the electrical Ohmic contact between the probe and the semiconductor. Thus, despite that the measurements were consistent among the samples, they might have presented a systematic difference with respect to the results obtained using other electrical testing methods.

Avrami function fits were performed by minimizing the least squares errors between the Avrami Equation (1) and the experimentally obtained surface coverage data, that is, we minimized the least squares error:

$$\begin{array}{c} \left(t_{0,\mathrm{opt}},k_{\mathrm{opt}},n_{\mathrm{opt}}\right)=\underset{\left(t_{0},k,n\right)}{\mathrm{argmin}}\sum_{k=1}^{K}{\left[\frac{y\left(t_{k}\right)-F\left(t_{k};t_{0},k,n\right)}{\sigma (t_{k})}\right]}^{2}, \end{array}$$

where $y(t_{k})$ denote the experimental values at measurement times $t_{k}$, *K* is the number of temporal snapshots, $\sigma (t_{k})$ are the corresponding measurement uncertainties (depicted as error bars in Figure 4), and $F(t_{k};t_{0},k,n)$ denote the Avrami function values at times $t_{k}$, with respect to the parameters $\left(t_{0},k,n\right)$, as defined in (1). The overall fitness is represented by the (minimized) least squares error, as stated in the last column of Table 1.

## 4. Conclusions

We investigated the changes caused by different conditions in the DPP deposition mechanisms on Si during the MD process by changing the solution concentration. The morphological data show that: (i) the molecule is deposited on the samples at different rates, and higher solution concentrations lead to a faster rise in the sample surface coverage; (ii) the nucleation rate shows the same behavior, as it is higher with increasing solution concentrations. Despite the higher deposition rate, the hierarchy of the sheet resistance of the cleaned samples at any given time is inverted with respect to the surface coverage: higher solution concentrations are associated with higher resistances. This behavior has been linked to the ratio of physisorbed vs. chemisorbed molecules present on the samples before the applied surface cleaning. By fitting the Avrami function onto the surface coverage data, the values of *n* are calculated, which suggests that the solution concentration influences the dynamics of the DPP deposition. These values of *n* are associated with the presence of a transient, physisorbed state of the molecules during deposition by using more concentrated deposition solutions. An explanation of the behavior of the Rs values has been proposed. The available space for chemisorption of the DPP molecules on the Si surface depends on the nuclei surface density at any given time. With a higher nucleation rate (and thus higher solution concentrations), the surface areas available for chemisorption are reduced, and as a consequence, the sheet resistance of the cleaned samples increases. These results will give better insight into solution-based molecular deposition processes and will allow for a better understanding of the MD process, as well as other technological processes based on molecule–surface interactions such as molecular electronics and molecular contact doping, especially for nano-architecture, hollow structures and devices based on nanoscale doped structures. Future work foresees investigation of the role of the molecules’ chemical and physical properties (molecular structure, steric footprint, etc.), substrate orientation, and—when applied to high-density nanostructures—their surface density.

## Figures and Tables

**Table 1:** Weighted least squares fitting parameters.

Percentage [%]	$t_{0}$ [min]	*k* [a.u.]	*n* [a.u.]	LS Error [a.u.]
5	6.53	68.18	3.86	9.70 × 10${}^{-5}$
10	0.00	64.45	4.94	8.59 × 10${}^{-4}$
15	11.50	15.73	6.66	5.99 × 10${}^{4}$

## Data Availability

Not applicable.
